# INSC Is a Prognosis-Associated Biomarker Involved in Tumor Immune Infiltration in Colon Adenocarcinoma

**DOI:** 10.1155/2022/5794150

**Published:** 2022-09-12

**Authors:** Su Zhaoran, Jia Weidong

**Affiliations:** ^1^Department of Gastrointestinal Surgery, People's Hospital of Tongling City, Tongling, Anhui, China; ^2^Department of Digestive Endoscopy, People's Hospital of Tongling City, Tongling, Anhui, China; ^3^Department of General Surgery, The First Affiliated Hospital of USTC, Division of Life Science and Medicine, University of Science and Technology of China, Hefei, Anhui, China

## Abstract

**Aims:**

The purpose of this study was to investigate the correlation of INSC gene with the level of immune infiltration and clinical prognosis in colon adenocarcinoma (COAD) patients.

**Materials and Methods:**

INSC expression profile data and clinicopathological information of COAD patients were downloaded from TCGA. Xiantao bioinformatics tool was used to analyze the expression of INSC between the COAD group and the normal control group, and GEPIA2 was used to analyze the top 100 coexpressed genes. Logistic regression analysis was performed to assess the relationship between clinicopathological features and INSC. The Kaplan-Meier method and Cox regression model were used to perform the survival analysis. CIBERSORT algorithm was used to analyze the relationship between INSC expression and immune infiltration cells.

**Results:**

The expression level of INSC in COAD was significantly downregulated. The result of logistic regression analysis confirmed that tumor stage was the final influencing factor of INSC expression. The overall survival rate of INSC in the high expression group was higher than that of the low expression group, and it was an independent risk factor of prognosis. Enrichment results indicated that INSC was enriched in the regulation of T-helper 2 cell differentiation pathway. Immune infiltration analysis showed that INSC expression was positively correlated with the B cell plasma, T cell CD4+ memory resting, activated myeloid dendritic cells, and eosinophils.

**Conclusions:**

Our study found that the expression of INSC was significantly downregulated in COAD, which regulated immune-infiltrating cells during cancer development and was associated with malignant progression in COAD patients.

## 1. Introduction

Colon adenocarcinoma (COAD) is one of the most common malignant tumors worldwide. According to the recent global cancer epidemiological survey, the incidence of COAD ranks third in the global cancer incidence [1]. Tumor recurrence and metastasis were the main causes of death in COAD patients [2, 3]. When patients developed distant metastasis or recurrence after surgical treatment, the median survival rate after relapse was only 13.3 months. The standard treatment for unresectable advanced or recurrent COAD is systemic chemotherapy and targeted therapy for now [4], but due to the lack of effective chemotherapy and targeted therapy, the prognosis of these patients is still poor. Therefore, the investigation for new tumor characteristic molecules is helpful to discover new therapeutic targets, so as to provide appropriate individualized treatment for tumor patients which is an important means to improve the prognosis.

Inscuteable (INSC) gene was first isolated and characterized in Drosophila. In Drosophila, neuroblasts divide asymmetrically into another neuroblast at the apical side and a smaller ganglion mother cell on the basal side. Cell polarization is precisely regulated by 2 apically localized multiprotein signaling complexes that are tethered by inscuteable, which regulates their apical localization [5, 6]. In human, the function of the INSC gene is still unclear, and the expression and mechanism of INSC in cancer are rarely reported.

In this study, by analyzing the microarray data obtained from The Cancer Genome Atlas (TCGA) database, we explored the expression, immune infiltration, and prognostic value of INSC in COAD and predicted the molecular mechanism of INSC in the occurrence and development of COAD.

## 2. Materials and Methods

### 2.1. INSC Expression in Cancer

Xiantao bioinformatics tool (https://www.xiantao.love/products) is an online visualized bioinformatics analysis tool that can analyze RNA expression data of tumor samples and normal samples shared by TCGA and Genotype-Tissue Expression (GTEx) projects. We first performed an unpaired analysis of INSC mRNA expression in 33 cancers including COAD whereas paired analysis of INSC expression in COAD.

### 2.2. Analysis of the Relationship between Clinicopathological Features and INSC

mRNA expression of INSC and clinical information of COAD patients including gender, age, pathological stage, tumor stage, lymph node status, metastasis, lymphatic invasion, and perineural invasion based on TCGA were analyzed by using R package. We first performed univariate analysis to investigate the differential expression of INSC according to clinicopathological features. Then, logistic regression analysis was performed to assess the relationship between clinicopathological features and INSC. Receiver operating characteristic (ROC) curve was used to assess the sensitivity and specificity of INSC for diagnosing COAD.

### 2.3. Analysis of the Prognostic Significance of INSC

We explored the survival of COAD patients which data was retrieved from the TCGA database. Firstly, the COAD patients with valid prognostic information including overall survival (OS) were grouped based on INSC median expression and analyzed by using the log-rank method. Then, we used Cox regression analysis to determine the risk factors for the prognosis of COAD patients. The variables in the multivariate analysis included gender, age, tumor stage, lymph node status, metastasis, lymphatic invasion, perineural invasion, and INSC expression.

### 2.4. Analyses of Genes Coexpressed with INSC

The “Similar Genes Detection” functional subset of GEPIA2 (http://gepia2.cancer-pku.cn/#index) was used to analyze the top 100 coexpressed genes of INSC in COAD. Then, we performed an enrichment analysis for the top 100 coexpressed genes by using the clusterProfiler package in R (version 4.0.3).

### 2.5. Evaluation of Tumor-Infiltrating Immune Checkpoints

Correlations between INSC expression and various infiltrating immune cell types were investigated by using CIBERSORT algorithm which is integrated in the immunedeconv package of R. Additionally, correlations between IGFBP7 expression and checkpoint-related genes including CD274, CTLA4, HAVCR2, LAG3, PDCD1, PDCD1LG2, TIGIT, and SIGLEC15 were also explored.

### 2.6. Statistical Analyses

SPSS 19.0 software was used to perform analysis. The independent sample *t*-test, paired *t*-test, and Mann-Whitney *U*-test were used for the comparison between the two groups of measurement sample. Logistic regression analysis was performed to assess the influencing factors of INSC. ROC curve was used to assess the sensitivity and specificity for diagnosing. Multivariate Cox regression and log-rank analysis were used to study the prognosis. *P* value < 0.05 was considered to be statistically significant.

## 3. Results

### 3.1. The Expression of INSC in COAD

**Figure 1:**
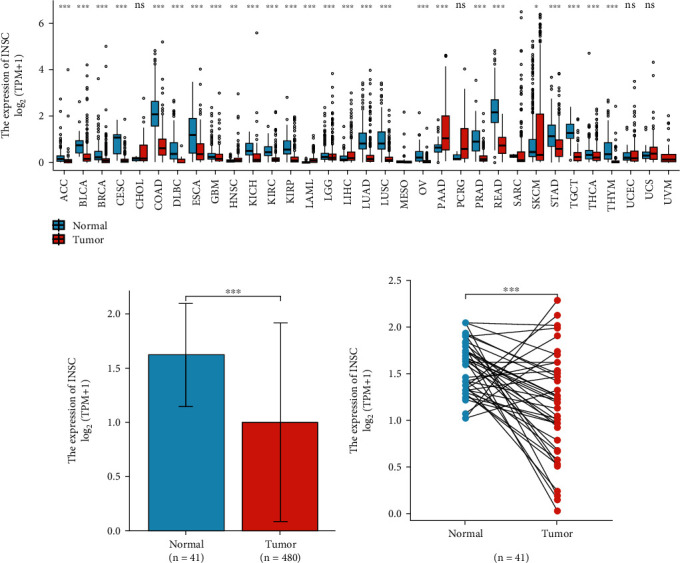
Expression difference of INSC in tumor (^∗^*P* < 0.05, ^∗∗^*P* < 0.001, and ^∗∗∗^*P* < 0.001). (a) INSC expression in 33 cancers which collected through TCGA and GTEx dataset. (b) Unpaired analysis of INSC expression between tumor and normal tissues collected through TCGA-COAD dataset. (c) Paired analysis of INSC expression between tumor and normal tissues collected through TCGA-COAD dataset.

The results of unpaired analysis based on data obtained from TCGA and GTEx as shown in Figure 1(a) confirmed that the expression of INSC in 33 tumors was divergent. Then, INSC expression profile data of 521 samples including cancerous and paracancerous tissue of COAD patients were downloaded from TCGA database and analyzed. The results of independent sample *t*-test (*P* < 0.001, Figure 1(b)) and paired *t*-test (*P* < 0.001, Figure 1(c)) analysis all confirmed that the expression of INSC was significantly downregulated in COAD.

### 3.2. Association between INSC Expression and Clinicopathological Variables

**Figure 2:**
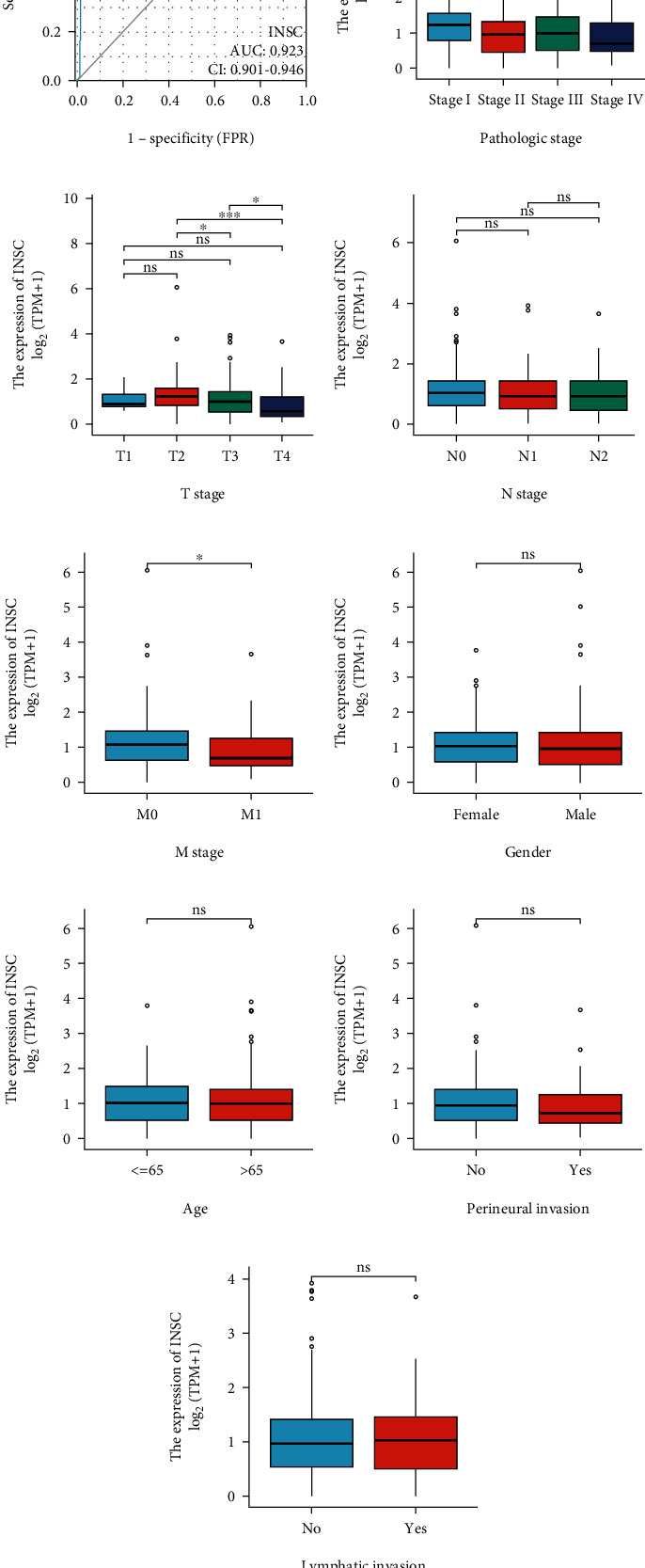
Association between INSC expression and clinicopathologic variables (^∗^*P* < 0.05, ^∗∗^*P* < 0.001, and ^∗∗∗^*P* < 0.001). (a) ROC curve shows the predictive ability of INSC for COAD (AUC = 0.923, CI: 0.901–0.946). Expression of INSC between different pathological stage (b), tumor stage (c), lymph node status (d), metastasis (e), gender (f), age (g), perineural invasion (h), and lymphatic invasion (i).

We used ROC curves to analyze the INSC expression data of 639 samples including cancerous and normal colon tissue received from TCGA and GTEx to evaluate the role of INSC in the diagnosis of COAD. The results showed that the sensitivity and specificity of INSC for the diagnosis of COAD were 0.838 and 0.905, respectively (Figure 2(a)). The area under the ROC curve (AUC) was 0.923 (CI: 0.901–0.946). Then, the data of 478 COAD cases with valid clinical information and INSC expression were downloaded by TCGA. Firstly, univariate analysis was performed to compare the differential expression of INSC according to clinicopathological features including pathological stage (Figure 2(b)), tumor stage (Figure 2(c)), lymph node status (Figure 2(d)), metastasis (Figure 2(e)), gender (Figure 2(f)), age (Figure 2(g)), perineural invasion (Figure 2(h)), and lymphatic invasion (Figure 2(i)). The results showed that INSC was downregulated in higher pathological stage (*P* < 0.01), tumor stage (*P* < 0.001), and metastasis groups (*P* < 0.05). Then, logistic regression analysis was performed to assess the relationship between clinicopathological features and INSC, and the result confirmed that tumor stage (*P* = 0.007) was the final influencing factor of INSC expression (Table 1).

### 3.3. Association between INSC Expression and Prognosis in COAD Patients

**Figure 3:**
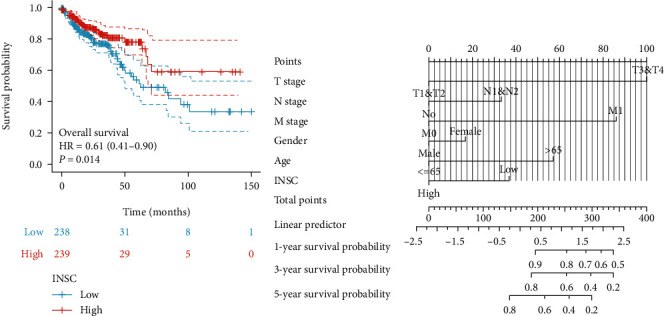
Association between INSC expression and prognosis. (a) The increased expression of INSC in COAD is associated with a favorable OS. (b) Multivariate Cox analysis of INSC expression and other clinicopathological factors.

Firstly, the survival package in R was used to analyze the OS of 477 COAD patients with valid prognostic information by using the log-rank method grouped based on INSC median expression. The results showed that increased expression of INSC in COAD is associated with a favorable prognosis (*P* = 0.014, Figure 3(a)). Then, we used Cox regression analysis to determine the risk factors for the prognosis of COAD patients. The variables in the multivariate analysis included gender, age, tumor stage, lymph node status, metastasis, and INSC expression, and the results showed that age (*P* = 0.004), tumor stage (*P* = 0.028), metastasis (*P* < 0.001), and INSC expression (*P* = 0.033) were the ultimate influencers of OS (Figure 3(b) and Table 2).

### 3.4. Genes Coexpressed with INSC and Enrichment Analysis

**Figure 4:**
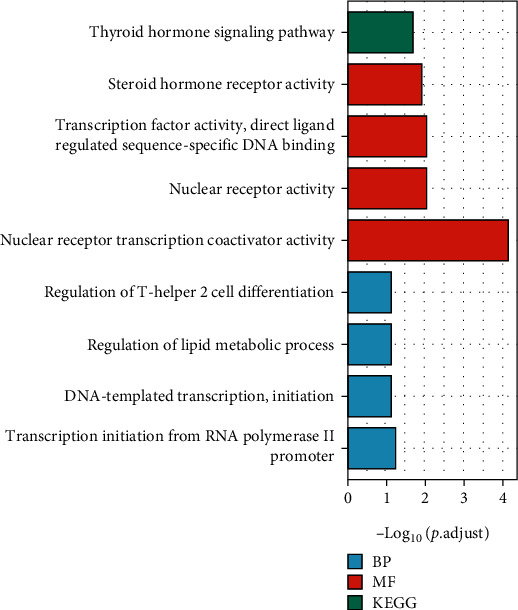
The top 100 coexpressed genes of INSC were selected to conduct the enrichment.

The top 6 genes coexpressed with INSC were RPS20P1 (PCC = 0.88), AMMECR1LP1 (PCC = 0.88), TMEM229A (PCC = 0.88), RP11-644F5.15 (PCC = 0.84), XXbac-BPG300A18.13 (PCC = 0.84), and CALCB (PCC = 0.83). The top 100 genes coexpressed with INSC were selected for enrichment analysis. The terms transcription initiation from RNA polymerase II promoter, DNA-templated transcription and initiation, regulation of lipid metabolic process, and regulation of T-helper 2 cell differentiation were significantly enriched in the GO term biological process (BP) analysis. In the GO term molecular function (MF) analysis, the terms nuclear receptor transcription coactivator activity, nuclear receptor activity, transcription factor activity, direct ligand regulated sequence-specific DNA binding, and steroid hormone receptor activity were highly enriched. The KEGG pathway analysis indicated that thyroid hormone signaling pathway was significantly enriched (Figure 4).

### 3.5. Association between INSC Expression and Tumor Immune Infiltration

**Figure 5:**
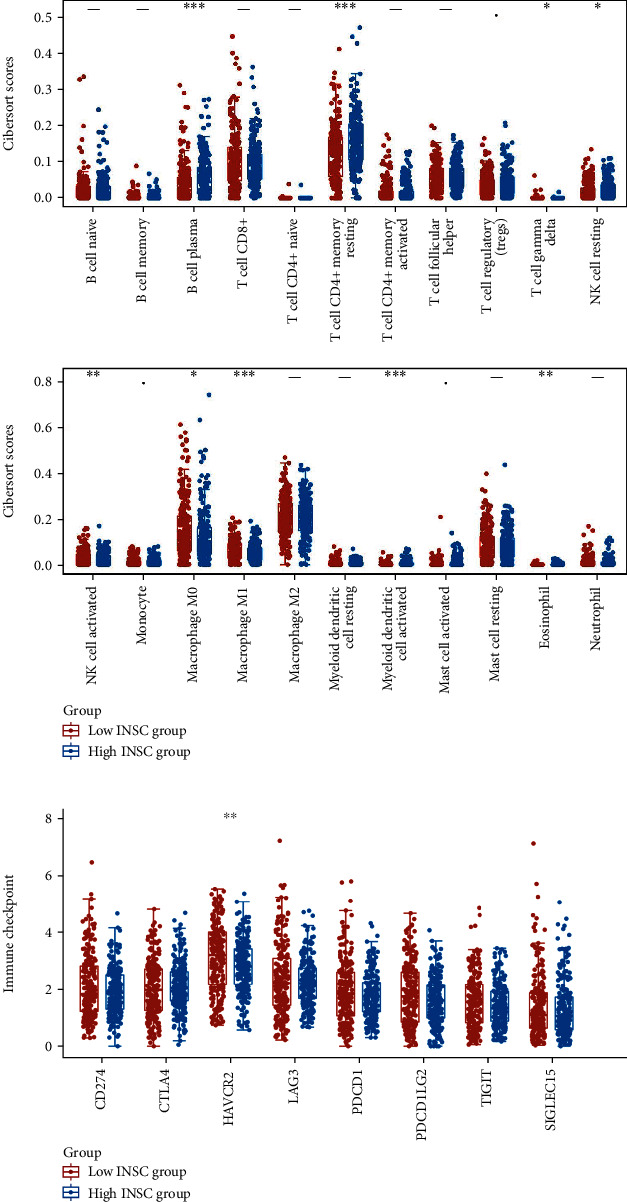
Relationship between INSC expression and tumor immune infiltration level (^∗^*P* < 0.05, ^∗∗^*P* < 0.001, and ^∗∗∗^*P* < 0.001). (a) Tumor-infiltrating immune cell levels between different INSC expression groups. (b) The expression levels of immune checkpoint-related genes between different INSC expression groups.

We analyzed the relationship between 22 immune infiltrate cell abundance and the expression of INSC in COAD. 521 COAD samples were divided into INSC high group and low group based on INSC median expression. Then, the abundance of immune infiltrates cells were analyzed by using CIBERSORT algorithm. The results showed that the B cell plasma (*P* < 0.001), T cell CD4+ memory resting (*P* < 0.001), activated myeloid dendritic cells (*P* < 0.001), and eosinophil (*P* < 0.001) were significantly higher, while the number of T cell gamma delta (*P* < 0.001), NK cell activated (*P* < 0.001), macrophage M0 (*P* < 0.001), and macrophage M1 (*P* < 0.001) were significantly lower in high INSC expression group (Figure 5(a)). We also analyzed the expression levels of immune checkpoint-related genes including CD274, CTLA4, HAVCR2, LAG3, PDCD1, PDCD1LG2, TIGIT, and SIGLEC15, and the results showed that HAVCR2 (*P* < 0.01) was significantly higher in the low INSC expression group (Figure 5(b)).

## 4. Discussion

Here, our investigation found that INSC was lowly expressed in COAD compared with normal tissues. Survival analysis found that the high expression of INSC was significantly correlated with poor overall survival in COAD, and the expression of INSC was related to COAD pathological stage, tumor stage, and metastasis. These findings suggest that INSC is a prognostic biomarker for COAD.

In mammals, the function of the INSC gene is still unclear and never reported in tumor. Therefore, we analyzed the genes coexpressed with INSC in COAD and performed an enrichment analysis for the top 100 coexpressed genes in this investigation. Our results showed that regulation of T-helper 2 cell differentiation was significantly enriched in the GO term BP analysis. This suggests that INSC may play a specific role in tumor microenvironment and tumor immunity. So we further investigated the correlation of INSC with immune infiltrating cells in COAD.

Another important finding of this study is that the expression of INSC is related to the level of immune infiltration cells in COAD. In our investigation, the expression of INSC was positively correlated B cell plasma, T cell CD4+ memory resting, activated myeloid dendritic cells, and eosinophil cells.

The host immune response is involved in the whole process of tumor development and growth. In the histopathological analysis of COAD, tumor-infiltrating lymphocytes (TILs) are often interpreted as host umbrellas against tumor development [7–9]. TILs mediate the recruitment, maturation, and activation of immune cells, thereby inhibiting tumor growth. Tumors express proteins with unique mutations not found in normal tissues [10, 11]. Some of these mutant proteins can trigger specific T lymphocyte responses and thus may be recognized as neoantigens. T lymphocytes play an important role in both innate and acquired immunity. The content of CD4+ T lymphocytes reflects the body's antitumor immunity to a certain extent. Cytotoxic T cells can kill tumor cells by secreting cytotoxic granules, thereby promoting tumor cell apoptosis, and at the same time, they can activate and kill tumor cells under the signal transduction of helper T cells. If the ratio decreases, the patient's immune function is in a low state, and the proliferation and spread of the tumor will be enhanced. Therefore, the infiltration of CD4+ T lymphocytes in adjacent tissues and the changes in peripheral blood can reflect the development trend of tumors to a certain extent [12–15]. In this study, we found that in COAD patients with high INSC expression, the levels of various immune lymphocytes increased, which may be an explanation for the better prognosis of these patients.

Immune checkpoints are inhibitory regulatory molecules in the immune system that are critical for maintaining self-tolerance, preventing autoimmune responses, and minimizing tissue damage by controlling the timing and intensity of immune responses [16–18]. Immune checkpoint inhibitors are some monoclonal antibodies developed for corresponding immune checkpoints. The main role is to block the interaction between tumor cells expressing immune checkpoints and immune cells, thereby blocking the inhibition effect of tumor cells on immune cells. We analyzed the expression levels of main immune checkpoint-related genes including CD274, CTLA4, HAVCR2, LAG3, PDCD1, PDCD1LG2, TIGIT, and SIGLEC15, and the result showed that HAVCR2 (*P* < 0.01) was significantly higher in the low INSC expression group which was with poor prognosis. The above results indicate that INSC can be used as a marker for immunotherapy in COAD.

In conclusion, the expression of NSC was significantly downregulated in COAD tissues, which regulated immune-infiltrating cells during cancer development and was associated with malignant progression in COAD patients. Through a more in-depth study of its function, it may be able to serve as an independent prognostic factor for COAD, as well as a new therapeutic molecular target.

## Figures and Tables

**Table 1:** Association between INSC expression and clinicopathologic variables.

Characteristics	Odds ratio	OR	*P* value
T stage (T3&T4 vs. T1&T2)	0.526	0.328-0.832	0.007
N stage (N1&N2 vs. N0)	0.731	0.506-1.054	0.094
M stage (M1 vs. M0)	0.607	0.353-1.030	0.067
Age (>65 vs. ≤65)	0.901	0.625-1.298	0.576
Gender (male vs. female)	0.845	0.590-1.211	0.360
Perineural invasion (yes vs. no)	0.521	0.253-1.038	0.069
Lymphatic invasion (yes vs. no)	1.114	0.757-1.640	0.585

**Table 2:** Cox proportional hazards model for overall survival.

Characteristics	Univariate analysis		Multivariate analysis	
	Hazard ratio (95% CI)	*P* value	Hazard ratio (95% CI)	*P* value
T stage				
T1&T2	Reference			
T3&T4	3.072 (1.423-6.631)	0.004	3.749 (1.152-12.195)	0.028
N stage				
N0	Reference			
N1&N2	2.592 (1.743-3.855)	<0.001	1.567 (0.938-2.619)	0.087
M stage				
M0	Reference			
M1	4.193 (2.683-6.554)	<0.001	3.004 (1.776-5.081)	<0.001
Gender				
Female	Reference			
Male	1.101 (0.746-1.625)	0.627		
Age				
≤65	Reference			
>65	1.610 (1.052-2.463)	0.028	2.040 (1.257-3.310)	0.004
INSC				
Low	Reference			
High	0.606 (0.406-0.905)	0.014	0.619 (0.398-0.962)	0.033

## Data Availability

This study is based on several public databases, and the relevant databases and hyperlinks cited are fully described in the manuscript.
